# Machine learning‐mediated identification of ferroptosis‐related genes in osteonecrosis of the femoral head

**DOI:** 10.1002/2211-5463.13764

**Published:** 2024-01-11

**Authors:** Xiaojing Huang, Hongming Meng, Zeyu Shou, Han Zhou, Liangyan Chen, Jiahuan Yu, Kai Hu, Zhibiao Bai, Chun Chen

**Affiliations:** ^1^ Department of Orthopedics The First Affiliated Hospital of Wenzhou Medical University China; ^2^ Wenzhou Medical University China; ^3^ Key Laboratory of Intelligent Treatment and Life Support for Critical Diseases of Zhejiang Province Wenzhou China; ^4^ Zhejiang Engineering Research Center for Hospital Emergency and Process Digitization Wenzhou China

**Keywords:** bioinformatics analysis, ferroptosis, immune infiltration, machine learning, osteonecrosis of the femoral head

## Abstract

Osteonecrosis of the femoral head (ONFH) is a condition caused by a disruption or damage to the femoral head's blood supply, which causes the death of bone cells and bone marrow components and prevents future regeneration. Ferroptosis, a type of controlled cell death, is caused by iron‐dependent lipid peroxidation. Here, we identified ferroptosis‐related genes and infiltrating immune cells involved in ONFH and predicted the underlying molecular mechanisms. The GSE123568 dataset was subjected to differential expression analysis to identify genes related to ferroptosis. Subsequently, GO and KEGG pathway enrichment analyses, as well as protein–protein interaction (PPI) network analysis, were conducted. Hub genes involved in ferroptosis were identified using machine learning and other techniques. Additionally, immune infiltration analysis and lncRNA–miRNA‐mRNA network prediction analysis were performed. Finally, we determined whether ferroptosis occurred by measuring iron content. The hub genes were validated by ROC curve analysis and qRT–PCR. Four ferroptosis‐related hub genes (MAPK3, PTGS2, STK11, and SLC2A1) were identified. Additionally, immune infiltration analysis revealed a strong correlation among ONFH, hub genes, and various immune cells. Finally, we predicted the network relationship between differentially expressed lncRNAs and hub genes in the lncRNA–miRNA–mRNA network. MAPK3, PTGS2, STK11, and SLC2A1 have been identified as potential ferroptosis‐related biomarkers and drug targets for the diagnosis and prognosis of ONFH, while some immune cells, as well as the interaction between lncRNA, miRNA, and mRNA, have also been identified as potential pathogenesis markers and therapeutic targets.

## Graphical abstract

We identified MAPK3, PTGS2, STK11, and SLC2A1 as ferroptosis‐related hub genes in osteonecrosis of the femoral head (ONFH) by machine learning and then verified them experimentally. Immune infiltration analysis revealed strong correlation among ONFH, hub genes, and various immune cells. Finally, we mapped the regulatory network between lncRNAs and the hub genes.

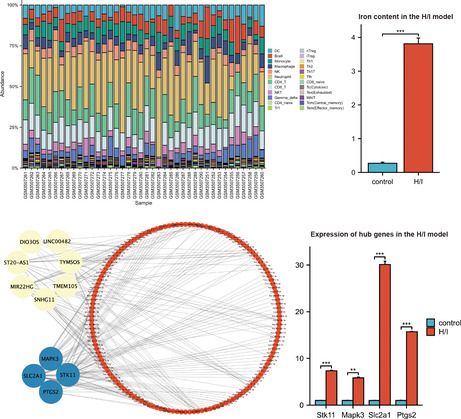

**Abbreviations**
AUC: area under curve
BMSCs: bone marrow mesenchymal stem cells
BP: biological process
CC: cellular component
DEGs: differentially expressed genes
GEO: Gene Expression Omnibus
GLM: generalized linear model
GO: Gene Ontology
H/I: Hypoxia/Ischemia
HGNC: HUGO Gene Nomenclature Committee
KEGG: Kyoto Encyclopedia of Genes and Genomes
LASSO: least absolute shrinkage and selection operator
MCC: maximal clique centrality
MF: molecular function
ONFH: osteonecrosis of the femoral head
PCA: principal component analysis
PPI: protein–protein interaction
qRT‐PCR: quantitative real‐time polymerase chain reaction
RF: random forest
ROC: receiver operating characteristic
SONFH: steroid‐induced osteonecrosis of the femoral head
SVM: support vector machines
UMAP: uniform manifold approximate projection
WB: western blot
XBG: eXtreme Gradient Boosting

Osteonecrosis of the femoral head (ONFH), also known as avascular necrosis of the femoral head, is a condition caused by a disruption or damage to the femoral head's blood supply, which causes the death of bone cells and bone marrow components and prevents future regeneration. One of the most common persistent disorders in the field of orthopedics is femoral head disease, which can cause structural abnormalities, collapse, and joint dysfunction. The disease's genesis can be split into the following two categories: traumatic and nontraumatic. The former is primarily caused by hip trauma, such as femoral neck fractures and hip dislocations; the latter is primarily caused by long‐term corticosteroid usage or long‐term drinking, smoking, and so on [1, 2, 3, 4].

Ferroptosis has received much interest from the scientific community in recent years. However, there have been few studies on the role of ferroptosis in ONFH. Ferroptosis, a type of controlled cell death, is caused by iron‐dependent lipid peroxidation, which can be induced or suppressed by pharmacological and genetic changes [5]. The term ferroptosis was very recently coined (2012); however, ferroptosis‐like cell death has long been reported, such as ‘oxytosis,’ an oxidative stress‐induced cell death. As research has advanced, there is increasing evidence that ferroptosis may play a physiological function in a range of illnesses. Ferroptosis is still a developing field of research that emerged from the fields of iron homeostasis, amino acid and lipid metabolism, redox and selenium biology, and cell death [6]. For the first time, we investigated ferroptosis‐related genes in ONFH using machine learning and other methodologies; these results may lead to new insights into ONFH and its treatment.

## Materials and methods

### Identification of DEGs

We used the dataset GSE123568 (platform: GPL15207, Affymetrix Human Gene Expression Array, Santa Clara, CA, USA) from the GEO database as the experimental dataset; GSE123568 included 10 blood samples from healthy humans and 30 blood samples from SONFH patients. The validation set was the GSE74089 dataset (platform: GPL13497, Agilent‐026652 Whole Human Genome Microarray 4x44K v2, Palo Alto, CA, USA).

We used r (version 3.6.3) for statistical analysis and visualization, downloaded datasets from the GEO database through the geoquery package (version 2.54.1) [7], and removed probes that correspond to multiple molecules. When a probe corresponding to the same molecule was encountered, only the probe with the largest signal value was retained, and then the data were normalized again by the normalizeBetweenArrays function of the limma package (version 3.42.2), New York, NY, USA; the sample normalization was checked by the box plot. The PCA chart and the UMAP chart were used to view the clustering between the sample groups, and then the difference analysis between the two groups was performed using the limma package.

In this study, we generated volcano plots by choosing adjusted *P* values < 0.05 and |log FC| values > 0.5 as criteria. The visualization was completed using the ggplot2 package (version 3.3.3), https://ggplot2.tidyverse.org. We used the complexheatmap package (version 2.2.0) [8] to visualize the top 20 genes with high and low expression. Then, ferroptosis‐related genes were obtained from the FerrDb database (www.zhounan.org/ferrdb), and ferroptosis‐related DEGs were obtained through Venn diagrams.

### GO and KEGG pathway enrichment analyses

We used the org.Hs.eg.db package (version 3.10.0) for ID conversion and the clusterprofiler package (version 3.14.3) for enrichment analysis [9]. The gene enrichment information included biological process (BP), cellular component (CC), molecular function (MF), and pathway enrichment information. Then, we performed analysis of the selected data with the clusterprofiler package (version 3.14.3) and visualization with the ggplot2 package (version 3.3.3).

### PPI network analysis

The selected ferroptosis‐related DEGs were used to construct a PPI network using the online analysis tool string (https://string‐db.org/) [10], and the minimum required interaction score was set to 0.400 (medium confidence); the protein–protein interaction data was then exported.

### The identification of hub genes

Based on the PPI network, the MCC algorithm in the cytoHubba plugin in cytoscape (version 3.7.3), Bethesda, MD, USA, was used to screen the hub genes, and the genes with the top 10 scores were obtained. We performed lasso regression analysis using the ‘glmnet’ package in R language to identify genes that were significant in discriminating ONFH samples from healthy specimens. Then, RF analysis, SVM analysis, XGB analysis, and GLM analysis were performed simultaneously. The top five results of RF analysis were selected through comprehensive evaluation of the residual and the area under the ROC curve, and the ferroptosis‐related hub genes were obtained by comparing with the results of lasso regression analysis and the MCC algorithm.

### Immune infiltration analysis

Osteonecrosis of the femoral head and normal samples were assessed and visualized using the ImmuCellAI algorithm (http://bioinfo.life.hust.edu.cn/ImmuCellAI#!/).

### Predictive analysis of the lncRNA–miRNA–mRNA network

The NetworkAnalyst platform (https://dev.networkanalyst.ca/NetworkAnalyst/home.xhtml) was used to predict the miRNA levels of the hub genes, and the miRTarBase v8.0 database was used for prediction. Then, we obtained all HGNC‐certified (HUGO Gene Nomenclature Committee) lncRNA gene names from the website (https://www.genenames.org/) and matched them with the DEGs we obtained above to identify differentially expressed lncRNAs. Afterward, we used the online platform DIANA‐LncBase v3 (https://diana.e‐ce.uth.gr/lncbasev3/interactions) for differential lncRNA–miRNA interaction identification with the conditions of ‘human’ and ‘high miRNA confidence level.’ Finally, cytoscape (version 3.7.3) was used for network visualization.

### ROC curve of hub genes

GSE74089 was obtained from the GEO database. After preliminary data processing, the r language ‘pROC’ package was used for analysis and the ggplot2 package was used for visualization.

### Hypoxia/ischemia(H/I) treatment

Hypoxia was achieved by using a CO_2_ water jacketed incubator (Baocheng, China), which could deplete the concentration of O_2_ down to 0.5%. Ischemic conditions were achieved by replacing the culture medium with serum and DMEM without glucose (Gibco, Thermofisher, Grand Island, NY, USA). BMSCs (GuangZhou Jennio Biotech Co., Ltd, Guangdong province, China) were placed in a CO_2_ water jacketed incubator for 24 h. Control plates were kept in normoxic conditions for the corresponding times.

### Testing for iron

Samples from the control group the H/I group were collected and lysed by sonication (35% intensity, open for 8 s and stopped for 5 s, totaling 2 min) and then placed at 4 °C for 30 min. Centrifugation was used to separate the supernatant (9000 ***g***, 30 min, 4 °C). Then, an iron assay was performed using an iron assay kit (Nanjing Jiancheng Bioengineering Institute, Jiangsu province, China, A039‐2‐1).

### qRT–PCR analysis

TRIzol was used to isolate total RNA from H/I cells. As a part of the cDNA synthesis process, reverse transcription was carried out at 65 °C for 5 min and 37 °C for 15 min. qRT–PCR was performed for 60 s at 95 °C, 40 cycles at 95 °C for 10 s, and 30 cycles at 60 °C. A formula for determining the relative expression level was constructed by deriving the target genes from ACTB (actin beta). The $2^{-\Delta \Delta C_{T}}$ method was used to calculate relative miRNA expression. Table 1 details the primers used in this study.

**Table 1:** Information of the primers' sequencing.

Gene	Forward primers	Reverse primers
*Mapk3*	TCCGCCATGAGAATGTTATAGGC	GGTGGTGTTGATAAGCAGATTGG
*Ptgs2*	TGAGCAACTATTCCAAACCAGC	GCACGTAGTCTTCGATCACTATC
*Stk11*	TTGGGCCTTTTCTCCGAGG	CAGGTCCCCCATCAGGTACT
*Slc2a1*	CAGTTCGGCTATAACACTGGTG	GCCCCCGACAGAGAAGATG
*Actb*	GGCAGCGGCAGGATACAC	TTCACAGGACACGAGCTG

### Statistical analysis

Student's *t*‐test was used to compare gene expression in OA specimens with that in healthy specimens. There was a statistically significant difference when ****P < 0.0001, ***P < 0.001, **P < 0.01, or *P < 0.05.

## Results

### Identification of DEGs

**Fig. 1:**
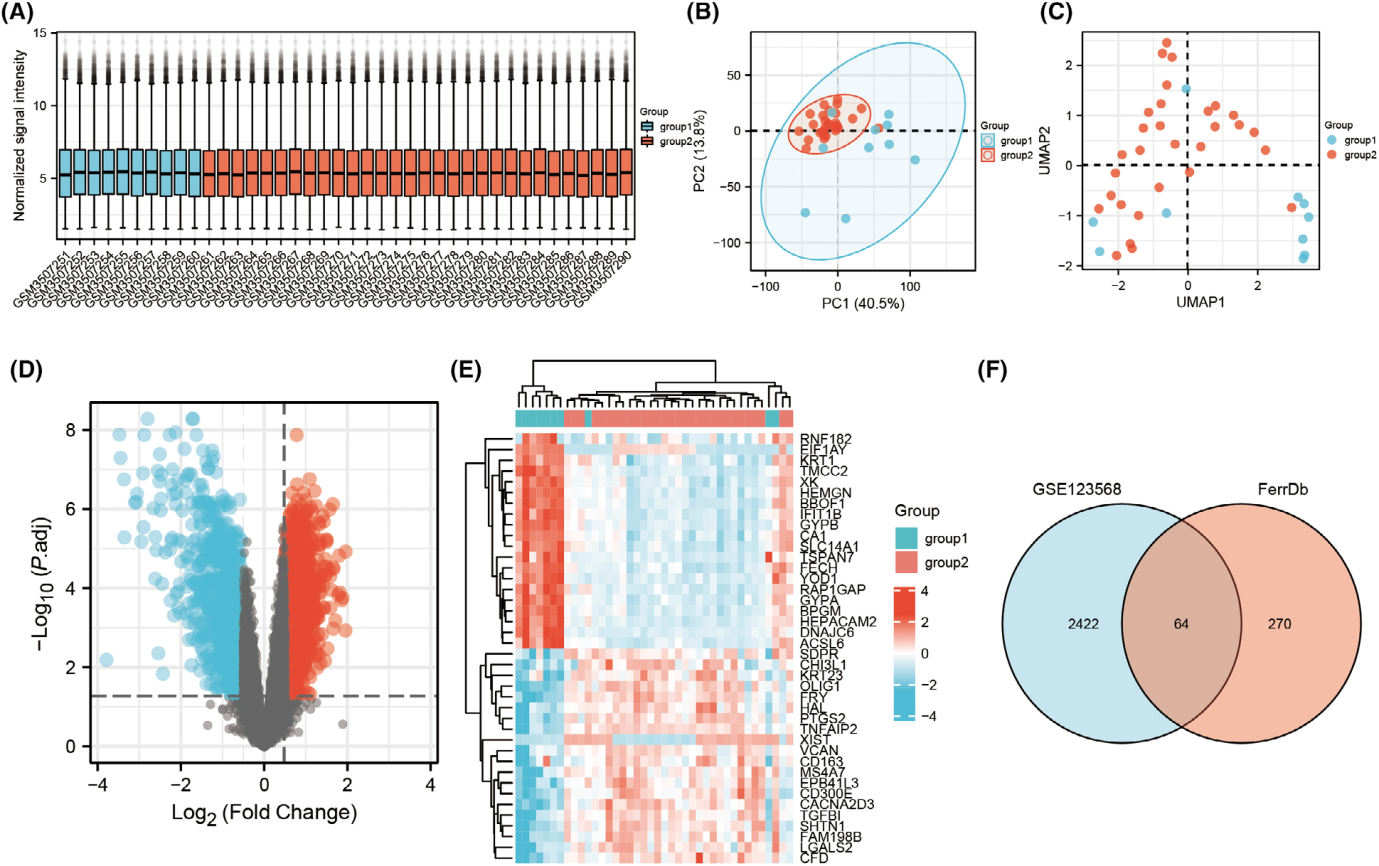
(A) Box plot after normalization of GSE123568. (B) PCA plot. (C) UMAP plot. (D) Volcano plot. (E) Heat map of expression of top20 genes with high and low expression. (F) Venn diagram. (Group 1 is healthy humans, and Group 2 is SONFH patients.).

The GSE123568 dataset was obtained by searching the keywords ‘necrosis of the femoral head’ in the GEO database. Through R language, the corresponding data were downloaded from the GEO database, and differential analysis was performed. Based on the box plot (Fig. 1A), the median of each sample was basically on a horizontal line, indicating that the degree of normalization between samples was good. The PCA graph (Fig. 1B) and the UMAP graph (Fig. 1C) showed that there were significant differences between the two groups. Afterward, the adjusted *P* value < 0.05 and the |log FC| value > 0.5 were used as the criteria for identifying DEGs and a volcano plot was generated; the volcano plot showed a large number of genes that fit the criteria. A total of 1469 upregulated DEGs and 1017 downregulated DEGs were obtained (Fig. 1D). Meanwhile, we selected the top 40 DEGs and created a heatmap, which also included some ferroptosis‐related genes (Fig. 1E).

We identified 334 ferroptosis‐related genes from the FerrDb database. By comparison with the above DEGs, we identified 64 ferroptosis‐related DEGs (Fig. 1F).

### KEGG/GO enrichment analyses

**Fig. 2:**
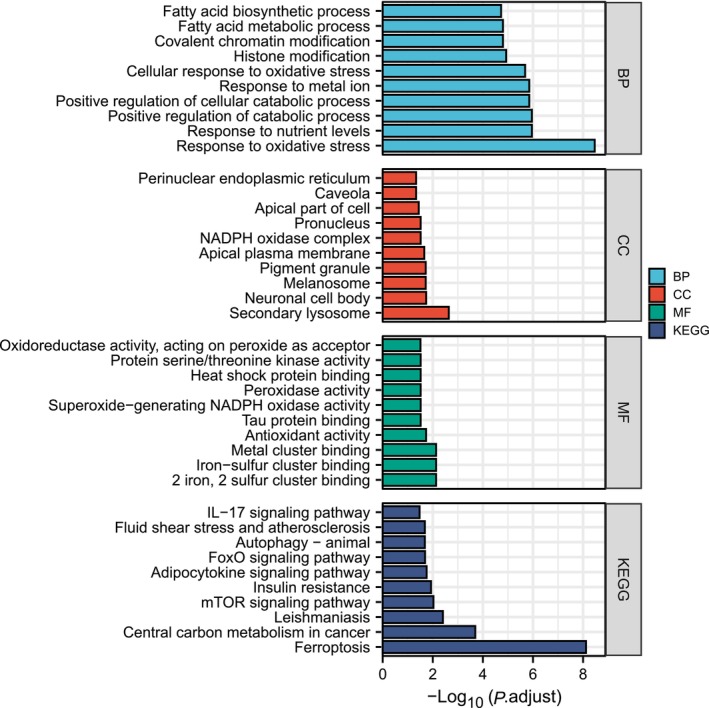
Top 10 results of GO/KEGG analysis of DEGs.

Under the conditions of *P*.adj < 0.05 and *q* value < 0.2, the enrichment results showed a total of 497 BPs, which mainly included antioxidant reactions, responses to nutrient levels, positive regulation of catabolic processes, and responses to metal ions. The CCs mainly focused on secondary lysosomes, neuronal cell bodies, melanosomes, pigment granules, and apical plasma membranes. The enriched MFs were iron–sulfur group binding, metal group binding, and antioxidant activity. KEGG pathway enrichment identified a total of 18 pathways, including ferroptosis (Table 2, Fig. 2).

**Table 2:** Top 5 results of GO/KEGG enrichment analysis of DEGs.

Ontology	ID	Description	GeneRatio	BgRatio	*P*‐value	*P*.adjust	*Q*‐value
BP	GO:0006979	Response to oxidative stress	16/63	451/18 670	1.34e‐12	3.42e‐09	2.16e‐09
BP	GO:0031667	Response to nutrient levels	14/63	499/18 670	8.92e‐10	1.10e‐06	6.96e‐07
BP	GO:0009896	Positive regulation of catabolic process	13/63	423/18 670	1.29e‐09	1.10e‐06	6.96e‐07
BP	GO:0031331	Positive regulation of cellular catabolic process	12/63	361/18 670	2.50e‐09	1.41e‐06	8.89e‐07
BP	GO:0010038	Response to metal ion	12/63	364/18 670	2.75e‐09	1.41e‐06	8.89e‐07
CC	GO:0005767	Secondary lysosome	3/63	14/19 717	1.10e‐05	0.002	0.002
CC	GO:0043025	Neuronal cell body	8/63	497/19 717	1.77e‐04	0.018	0.013
CC	GO:0042470	Melanosome	4/63	106/19 717	3.68e‐04	0.019	0.014
CC	GO:0048770	Pigment granule	4/63	106/19 717	3.68e‐04	0.019	0.014
CC	GO:0016324	Apical plasma membrane	6/63	318/19 717	5.27e‐04	0.022	0.016
MF	GO:0051537	2 iron, 2 sulfur cluster binding	3/63	22/17 697	6.31e‐05	0.007	0.006
MF	GO:0051536	Iron–sulfur cluster binding	4/63	63/17 697	7.42e‐05	0.007	0.006
MF	GO:0051540	Metal cluster binding	4/63	63/17 697	7.42e‐05	0.007	0.006
MF	GO:0016209	Antioxidant activity	4/63	86/17 697	2.49e‐04	0.019	0.015
MF	GO:0048156	Tau protein binding	3/63	45/17 697	5.48e‐04	0.030	0.024
KEGG	hsa04216	Ferroptosis	8/45	41/8076	4.02e‐11	7.60e‐09	5.68e‐09
KEGG	hsa05230	Central carbon metabolism in cancer	6/45	70/8076	2.13e‐06	2.01e‐04	1.50e‐04
KEGG	hsa05140	Leishmaniasis	5/45	77/8076	6.27e‐05	0.004	0.003
KEGG	hsa04150	mTOR signaling pathway	6/45	155/8076	1.99e‐04	0.009	0.007
KEGG	hsa04931	Insulin resistance	5/45	108/8076	3.11e‐04	0.012	0.009

### Construction of the PPI network and the identification of ferroptosis‐related hub genes

**Fig. 3:**
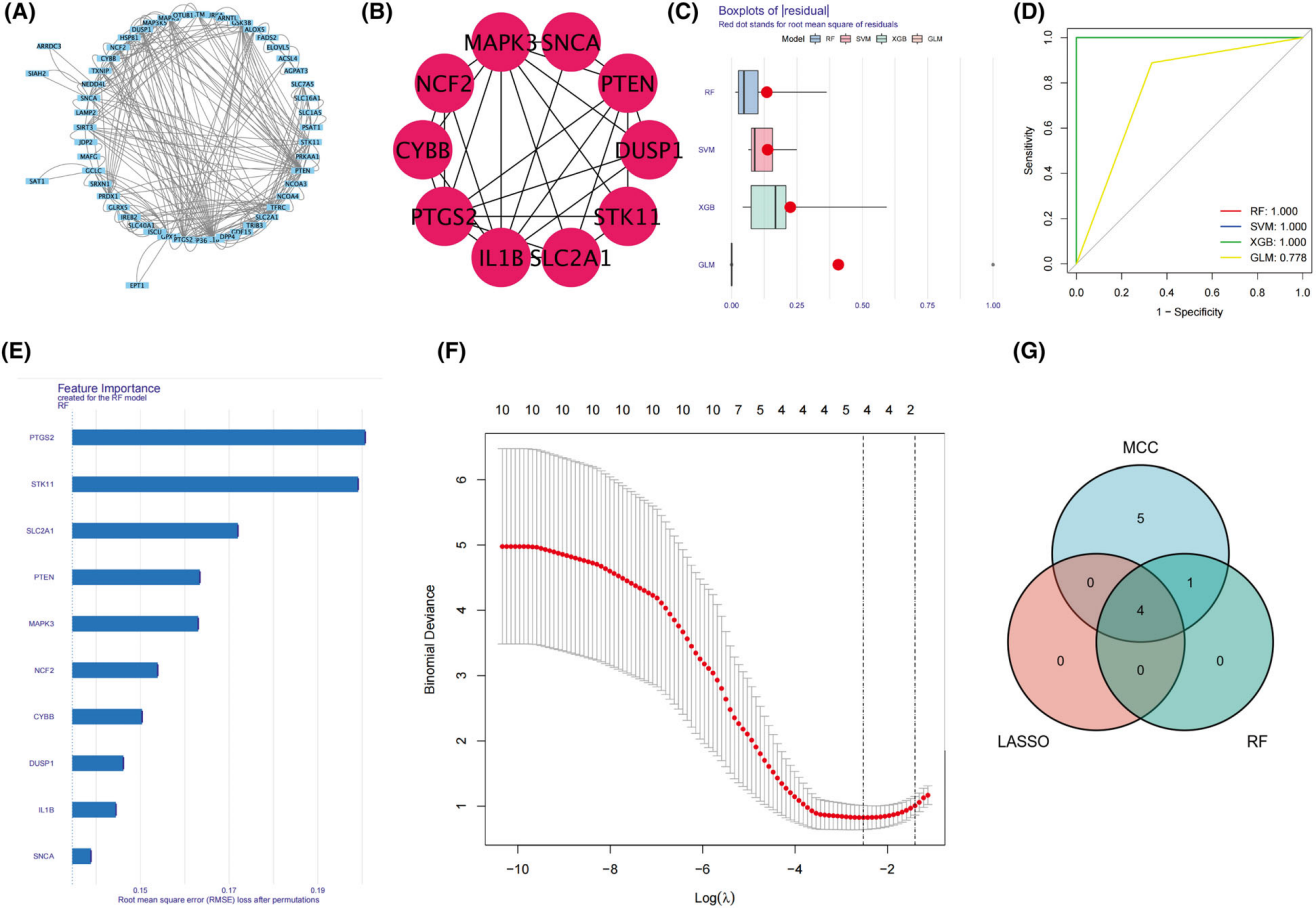
(A) PPI network. (B) Top 10 genes obtained by MCC algorithm. (C) The residual values of RF analysis, SVM analysis, XGB analysis and GLM analysis. (D) The area under the ROC curve of RF analysis, SVM analysis, XGB analysis and GLM analysis. (E) The rank of genes in RF. (F) LASSO regression analysis. (G) Venn of hub genes.

The PPI network was visualized using the cytoscape software (Fig. 3A), and the top 10 genes IL1B, MAPK3, PTGS2, STK11, SLC2A1, NCF2, CYBB, PTEN, DUSP1, and SNCA were obtained using the MCC algorithm feom the cytoHubba plugin (Table 3, Fig. 3B). By comparing the residual values and the area under the ROC curve of RF analysis, SVM analysis, XGB analysis and GLM analysis, we selected RF analysis and determined the top five genes (Fig. 3C–E). Then, we identified four significant genes by LASSO regression analysis (Fig. 3F). Finally, we compared the results to obtain the final ferroptosis‐related hub genes MAPK3, ATK11, PTGS2, and SLC2A1 (Fig. 3G).

**Table 3:** Top 10 ranked genes obtained by MCC algorithm.

Rank	Gene
1	*IL1B*
2	*MAPK3*
3	*PTGS2*
4	*STK11*
5	*SLC2A1*
6	*NCF2*
7	*CYBB*
8	*PTEN*
9	*DUSP1*
10	*SNCA*

### Immune cell infiltration analysis

**Fig. 4:**
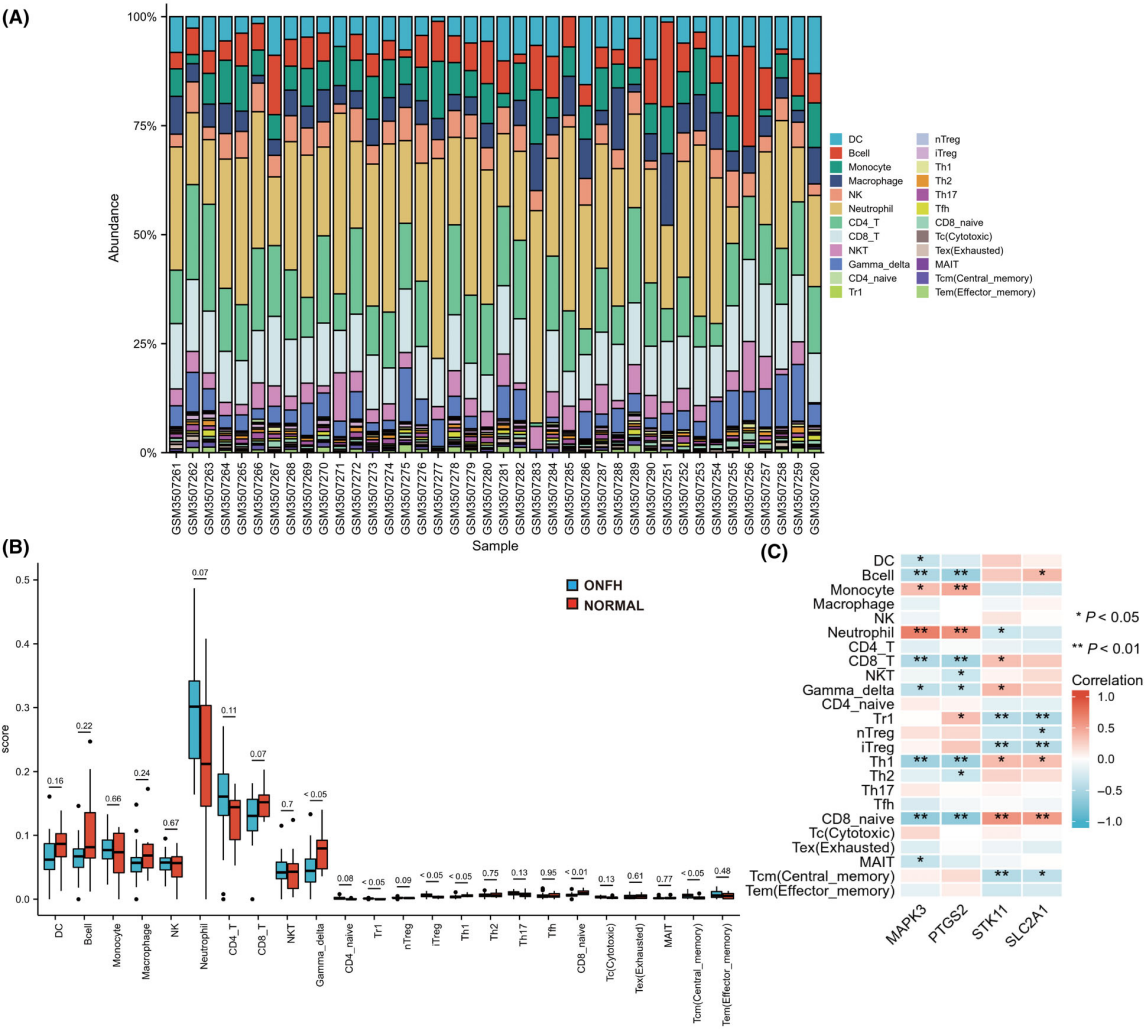
(A) Relative percentage of immune cell subsets in each sample. (B) Differences in immune cell infiltration between ONFH patients and normal controls (using Wilcoxon rank sum test and error bars represent the SEM). (C) Map of the correlation between Hub genes and immune cells. *P < 0.05; **P < 0.01; ***P < 0.001. Healthy samples = 10 and SONFH samples = 30.

Using the ImmuCellAI algorithm, we investigated the differences in the infiltration of 24 immune cell subsets between the two groups of samples in GSE123568, and the results showed that there was a significant difference in the proportion of immune cells between the two groups of samples (Fig. 4A). Compared with healthy samples, naive CD8 T cells, Th1 cells and Tgd cells in ONFH patients were significantly decreased, and Tcm cells, Tr1 cells and iTreg cells were significantly increased (Fig. 4B). The four hub genes were highly correlated with immune cell levels (Fig. 4C).

### Prediction of the lncRNA–miRNA–mRNA network

**Fig. 5:**
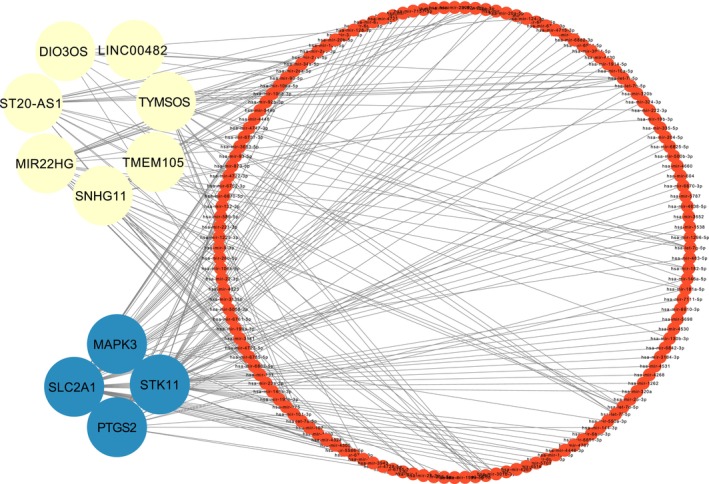
LncRNA‐miRNA‐mRNA network. Among them, lncRNAs are shown in yellow, miRNAs in red, and mRNAs in blue.

A total of 115 miRNAs were predicted to act on the hub genes via the NetworkAnalyst Website (Table 4, Fig. 5). A total of 5552 HGNC‐certified lncRNA genes were obtained from the online website and matched with DEGs to obtain 10 differentially expressed lncRNAs, namely *ASAP1‐IT2*, *DIO3OS*, *LINC00482*, *LINC00570*, *LINC01013*, *MIR22HG*, *SNHG11*, *ST20‐AS1*, *TMEM105*, and *TYMSOS*. Then, we used the online platform DIANA‐LncBase v3 to predict the interaction of differentially expressed lncRNAs with the previously predicted miRNAs (Table 4, Fig. 5). We used the cytoscape software to visualize the lncRNA–miRNA–mRNA network (Fig. 5).

**Table 4:** Number of predicted miRNAs.

mRNA	miRNA quantity	lncRNA	miRNA quantity
*MAPK3*	7	*DIO3OS*	8
*PTGS2*	20	*LINC00482*	2
*STK11*	38	*MIR22HG*	22
*SLC2A1*	50	*SNHG11*	19
		*ST20‐AS1*	17
		*TMEM105*	2
		*TYMSOS*	17
		*ASAP1‐IT2*	0
		*LINC00570*	0
		*LINC01013*	0

### Verification of hub genes and ferroptosis

**Fig. 6:**
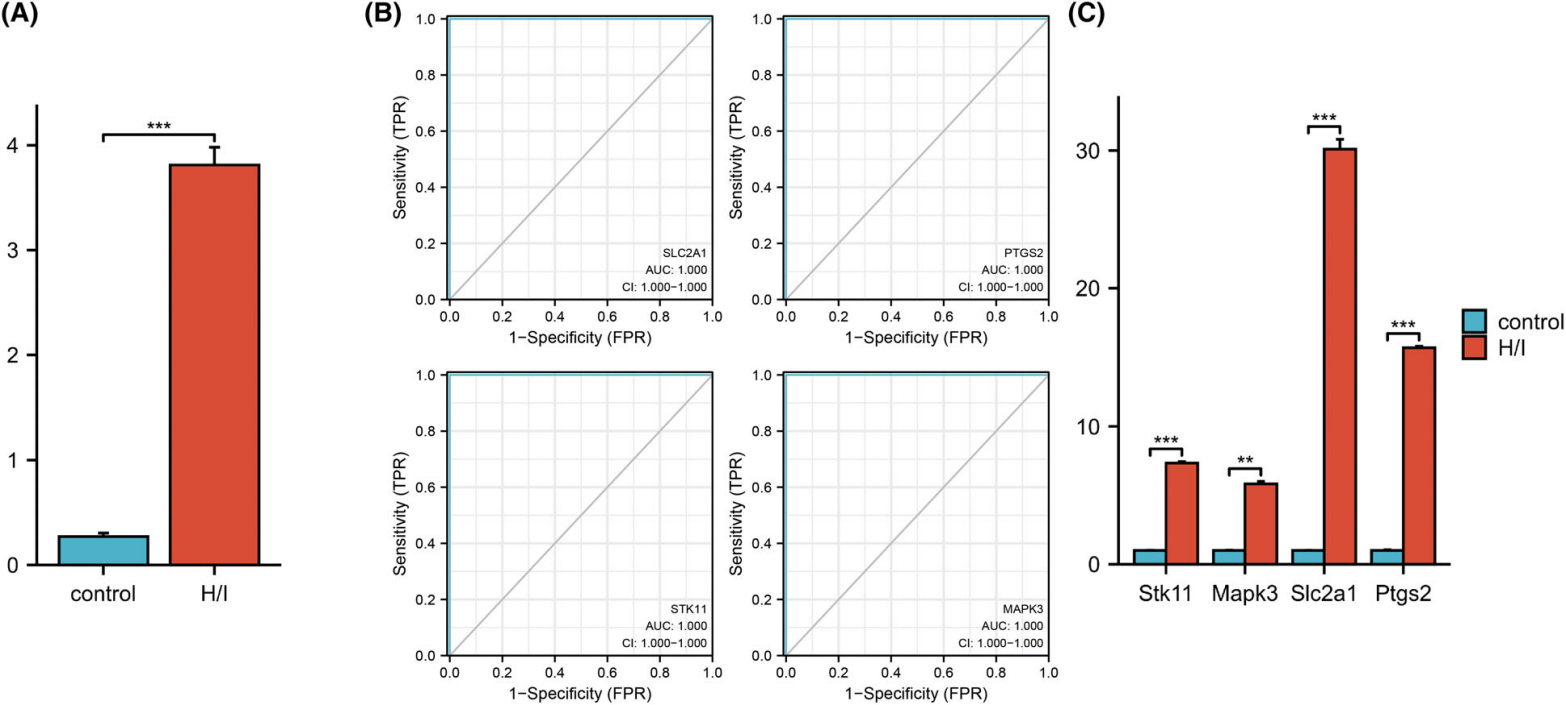
(A) Iron expression in control and H/I (using Student's *t*‐test). (B) ROC curves of four hub genes. (C) Expression of hub genes in the H/I model of BMSCs (using Student's *t*‐test). *P < 0.05; **P < 0.01; ***P < 0.001.

Our research revealed that ferroptosis occurred in the H/I group, where iron expression was much higher than that in the control group (Fig. 6A). We conducted ROC curve validation of the GSE74089 dataset to further assess the diagnostic effectiveness of the four hub genes, which showed that all four hub genes had an AUC of 100, and the findings demonstrated that they all had good diagnostic efficiency, highlighting the diagnostic utility of these markers for ONFH (Fig. 6B). At the same time, we used qRT–PCR to verify the hub genes, and the results indicated that the expressions of these genes were all upregulated, and the difference was statistically significant (Fig. 6C).

## Discussion

Osteonecrosis of the femoral head is a disabling disease. Currently, numerous academics are investigating its potential mechanism in terms of molecular biology [1, 11, 12]. However, the pathophysiology of this condition is still unknown, which makes it difficult to design therapeutic and preventive measures that are based on relevant mechanisms. In this paper, bioinformatics methods were used to study the differential expression in the peripheral serum of ONFH patients and healthy controls. Through analysis of GSE123568, we identified genes with potential importance in ONFH. We identified four ferroptosis‐related hub genes in peripheral blood samples. These genes were mainly involved in the ferroptosis signaling pathway. These hub genes might be important biomarkers related to ONFH pathogenesis and progression. Then, we used hip cartilage tissue samples (GSE74089) to verify that these four genes have high diagnostic value for ONFH. Meanwhile, we employed the ImmuCellAI tool to identify immune cell infiltration in ONFH. The results showed significant differences in immune cell infiltration between ONFH and control samples. In addition, we also predicted some lncRNA–miRNA–mRNA relationships associated with these potentially important genes. Finally, there was evidence of ferroptosis in the H/I group. Expression of the hub genes was confirmed by qRT–PCR, and the outcomes matched those of our analysis.

New types of cell death have been discovered in recent years, including ferroptosis. Various factors can reduce glutathione peroxidase's antioxidant capacity and cause lipid reactive oxygen species to accumulate in cells directly or indirectly, ultimately leading to oxidative cell death [13, 14]. The DEGs screened in this study can help clarify the role of ferroptosis‐related molecules or pathways in ONFH. These new molecules or pathways can be potentially important targets for disease diagnosis and treatment.

These four important ferroptosis‐related genes and their expression were validated by qRT–PCR, and the results were ideal. *PTGS2*, also known as *COX2*, is mainly involved in inflammation and prostaglandin synthesis. Previous studies have shown that *COX2* can promote osteoclast differentiation and migration through prostaglandin E2 [15], and *COX2* is an important marker of ferroptosis [16, 17]. *MAPK3*, also known as *ERK1*, has been reported to promote ferroptosis through its signaling pathway [18, 19]. However, the specific role of *MAPK3* and its pathway in the ferroptosis process in ONFH needs to be further studied. *SLC2A1* is a facilitative glucose transporter that is responsible for constitutive or basal glucose uptake [20, 21, 22]. It has been suggested that high expression of *SLC2A1* is associated with ferroptosis [23]. *SLC2A1* regulates the metabolism and osteogenic ability of osteoblasts by mediating glucose transport [24, 25]. Previous studies suggest that *SLC2A1* may be involved in ONFH through ferroptosis. *STK11* is also called *LKB1*. Li et al. [26] showed that the LKB1‐AMPK axis can negatively regulate ferroptosis by inhibiting fatty acid synthesis. Studies have also shown that the loss of *STK11* can inhibit the osteogenic ability of BMSCs, leading to high bone turnover, cortical porosity, and increased trabecular bone density [27, 28]. Our results showed that *STK11* expression was increased in the H/I model, which may be related to negative feedback regulation by cells themselves due to apoptosis triggered by ischemia‐hypoxia. Therefore, strengthening the role of *STK11* in early ONFH may delay or prevent the further development of ONFH. In conclusion, our study showed that these four ferroptosis‐related genes are associated with ONFH. Considering that these genes are associated with ferroptosis and that *PTGS2*, a characteristic ferroptosis gene, was also significantly increased in the ONFH group, we can infer that these genes may play a role in ONFH to some extent through ferroptosis.

We also performed immune infiltration analysis of the data, and the results showed that there were significant differences in the abundance of several immune cells, such as naive CD8 T cells, Th1 cells, Tgd cells, Tcm cells, Tr1 cells, and iTreg cells. At the same time, we explored the relationship between hub genes and immune cells, showing that hub genes are associated with a variety of immune cells, such as naive CD8 T cells and Th1 cells. In addition, some studies have shown that an imbalance in T cells may promote ONFH, but the specific mechanism is still unclear, and further research and exploration are needed [29, 30, 31]. Finally, we analyzed 10 differentially expressed lncRNAs, predicted the miRNAs of the hub genes and differentially expressed lncRNAs, and predicted a lncRNA–miRNA‐mRNA network. However, determining their specific roles and regulatory relationships in ONFH will require further research.

At present, there are still no good methods to prevent the occurrence of ONFH. When ONFH develops early, the use of anticoagulation therapy combined with vasodilator drugs can be recommended. However, when ONFH is severe, surgery is often needed, such as core decompression and joint replacement [31]. We identified 64 ferroptosis genes from molecular biological analysis. These genes are expected to become new avenues for ONFH mechanism research and may also become targets for new treatment methods, especially the four genes *MAPK3*, *PTGS2*, *STK11*, and *SLC2A1* that were screened and validated. Moreover, the lncRNA–miRNA–mRNA network, immune aspects, and signaling pathways of hub genes may also be potential targets for the treatment of ONFH.

Although several key hub genes, types of infiltrating immune cells, and potential lncRNA–miRNA–mRNA relationships were identified in our study, there are some limitations. First, our study was not validated *in vivo* and lacked WB validation. Second, our experiments did not specifically investigate the pathways through which these genes participate in the occurrence and development of ferroptosis in ONFH. However, our study still provides new insights into the genes involved in ferroptosis in ONFH.

## Conclusion

We identified *MAPK3*, *PTGS2*, *STK11*, and *SLC2A1* as ferroptosis‐related hub genes in ONFH for the first time by a machine learning method and verified them experimentally. Meanwhile, we performed immune infiltration analysis, which indicated that naive CD8 T cells, Th1 cells, etc., were significantly different between ONFH samples and healthy group samples and highly correlated with ferroptosis‐related hub genes. Finally, we identified 10 differentially expressed lncRNAs in ONFH, including *ASAP1‐IT2*, *DIO3OS*, *LINC00482*, *LINC00570*, *LINC01013*, *MIR22HG*, *SNHG11*, *ST20‐AS1*, *TMEM105*, and *TYMSOS*, and predicted their lncRNA–miRNA–mRNA network relationship with hub genes. These ferroptosis‐related genes, immune cells, and related networks may be involved in the occurrence and development of ONFH through ferroptosis, and they can be used as new avenues for pathogenic mechanism research and research on potential therapeutic targets for ONFH.

## Conflict of interest

The authors declare no conflict of interest.

## Author contributions

XH, HM, ZB, and CC conceived and designed the project. XH and HM acquired the data. XH, HM, LC, and JY analyzed and interpreted the data. HZ, ZS, and KH mainly carried out cell experiments and mapping. XH and HM wrote the paper. All the authors reviewed the manuscript.

## Data Availability

The datasets generated and analyzed during this study are available in the GEO repository (https://www.ncbi.nlm.nih.gov/geo/).
